# Nothing magical: pantomimed grasping is controlled by the ventral system

**DOI:** 10.1007/s00221-016-4868-1

**Published:** 2017-03-15

**Authors:** Thijs Rinsma, John van der Kamp, Matt Dicks, Rouwen Cañal-Bruland

**Affiliations:** 1 Research Institute MOVE Amsterdam, Faculty of Behavioural and Movement Sciences, VU University Amsterdam, Van der Boechorststraat 9, 1081 BT Amsterdam, The Netherlands; 2 Institute of Human Performance, University of Hong Kong, Hong Kong SAR, China; 3 Department of Sport and Exercise Science, University of Portsmouth, Portsmouth, UK; 4 Institute of Sport Science, Friedrich-Schiller-University Jena, Jena, Germany

**Keywords:** Pantomime, Two-visual systems, Grasping, Allocentric information, Visual illusion

## Abstract

In a recent amendment to the two-visual-system model, it has been proposed that actions must result in tactile contact with the goal object for the dorsal system to become engaged (Whitwell et al., Neuropsychologia 55:41–50, 2014). The present study tested this addition by assessing the use of allocentric information in normal and pantomime actions. To this end, magicians, and participants who were inexperienced in performing pantomime actions made normal and pantomime grasps toward objects embedded in the Müller–Lyer illusion. During pantomime grasping, a grasp was made next to an object that was in full view (i.e., a displaced pantomime grasping task). The results showed that pantomime grasps took longer, were slower, and had smaller hand apertures than normal grasping. Most importantly, hand apertures were affected by the illusion during pantomime grasping but not in normal grasping, indicating that displaced pantomime grasping is based on allocentric information. This was true for participants without experience in performing pantomime grasps as well as for magicians with experience in pantomiming. The finding that the illusory bias is limited to pantomime grasping and persists with experience supports the conjecture that the normal engagement of the dorsal system’s contribution requires tactile contact with a goal object. If no tactile contact is made, then movement control shifts toward the ventral system.

## Introduction

Milner and Goodale (1995, 2008, see also Goodale and Milner 1992) have posited that the visual brain is organised into two functionally distinct neuroanatomical systems: a ventral system for perception and a dorsal system for action. According to this two-visual-system hypothesis, perception and action place different functional demands on the visual system, and hence, the two systems can be distinguished by the types of information they preferably exploit. While perception requires enduring information about (the properties of) persons, objects, events and places typically in relation to each other, action requires this information instantaneously and typically in relation to the body or action system. Consequently, the ventral and dorsal systems are dispositioned to use allocentric information (i.e., world-centred) and egocentric information (i.e., body-centred), respectively.^1^

A plethora of neurobehavioural and physiological observations has supported Milner and Goodale’s (1995, 2008) two-visual-system hypothesis. At the behavioural level, the visual illusion paradigm has not only been the most pervasive body of evidence, but arguably also the most contentious (Bruno and Franz 2009; Ganel et al. 2008; Stöttinger et al. 2010; cf.; Franz and Gegenfurtner 2008; Smeets and Brenner 2006). In brief, the size of an object presented against an illusory background (e.g., a disc embedded in a Titchener illusion or a rod in a Müller–Lyer illusion) appears different than its real size, because the perception of an object is affected by its visual surroundings. However, when grasping an object, the unfolding hand aperture remains (relatively) unaffected by the illusory background, which is taken to demonstrate that actions are based on information that specifies an object’s real size. Nevertheless, when a delay is introduced between viewing the object and the execution of the grasp, the hand aperture is affected by the illusion (Westwood et al. 2001; Westwood and Goodale 2003). Thus, actions engage the dorsal system, but only when visual control is instantaneous, otherwise the ventral system intrudes.

Recently, Whitwell, Milner, Cavina–Pratesi, Byrne and Goodale (2014, see also Whitwell and Buckingham 2013; Willingham 1998) have added another prerequisite for the (normal) engagement of the dorsal system in action: the action must result in tactile contact with the goal object; otherwise it will recruit ventral system contributions. This addition to the two-visual-system hypothesis was prompted by observations of patient DF making grasps towards objects of different size in a mirror apparatus (Schenk 2012). Patient DF shows deficits in visual form perception in line with structural damage of her ventral stream (Bridge et al. 2013). Yet, her control of action towards an object remains largely accurate. Accordingly, Schenk (2012) showed that DF’s grasping in a mirror apparatus was indeed normal, but patient DF only demonstrated grip scaling if she received haptic feedback through contact with the object (see Whitwell et al. 2014). Presumably, grip scaling occurs as long as the contacted (unseen) object and the object viewed virtually in the mirror spatially coincide; their sizes, however, need not be congruent (Whitwell et al. 2015b).

These observations are reminiscent of an earlier report by Goodale, Jakobson and Keiller (1994) that patient DF was incapable of grip scaling when she was required to perform a pantomime grasp to a remembered object; that is, a grasp toward a location at which an object was initially presented but then removed during a 2 s interposition between viewing the object and the initiation of the grasping movement. It has been argued that this breakdown of action control results from DF’s damaged ventral system being incapable of replacing the dorsal system (see Whitwell et al. 2014, 2015a). In other words, without proper haptic contact, the dorsal system’s functioning in action gets disrupted.

However, a pantomime grasp for a remembered object does not only preclude haptic contact, but also prevents instantaneous access to visual information about object size. Accordingly, patient DF’s grip scaling remained largely accurate during a variant of the pantomime task—the real-time displaced pantomime grasp (Whitwell et al. 2015a). In this variant, DF was required to produce a grasping action at a location next to an object that was in full view (Goodale et al. 1994). Patient DF’s grip scaling was affected but not completely lost in this displaced pantomime task. Whitwell et al. (2014, 2015a) contended that the normal control by the dorsal system is interrupted when no haptic feedback is obtained from contact with the goal object. Under these circumstances, the ventral system is presumed to take over the control of action. Patient DF, however, managed to circumvent ventral system contributions during displaced pantomime grasping by using the table surface as a substitute object for making contact (cf. Schenk 2012).

We are interested in testing the claim that an action that does not result in proper contact with the goal object, such as in pantomime grasping, must—by default—engage the ventral system. Notably, the empirical evidence supporting this premise is largely indirect and stems chiefly from observations of only one neurological patient. That is, previous work demonstrated that without tactile contact, normal control gets disrupted, but it has not been proven that the ventral system takes over control of action. Moreover, since damage may disrupt the visual system’s functioning in an atypical manner (Bridgeman 2002), furthering our understanding of the visual control of actions that do not result in tactile contact requires additional investigation with healthy adults. Hence, we employed the visual illusion paradigm to examine whether pantomimed grasps in healthy adults do indeed engage the ventral system.

Westwood, Chapman and Roy (2000) examined pantomime grasping of healthy adults toward objects embedded in a Müller–Lyer illusion. In contrast to normal grasps, the grip aperture of the pantomime grasps was systematically affected by the illusory context, indicating that the ventral system was involved in the control of grasping. It is pertinent, however, that Westwood et al. (2000) used a delayed pantomime task (Whitwell et al. 2015a), in which the object is shown and removed, before performing the grasp. Participants thus grasped for remembered objects. However, precluding the online exploitation of visual information about the object also invokes ventral system engagement if the object is contacted (Westwood et al. 2001). Hence, to be able to attribute ventral engagement to a lack of contact with the goal object, we adopted the real-time displaced pantomime task, during which participants make a grasp next to the object that remains in full view during the action (see also Cavina–Pratesi et al. 2011).

Displaced pantomime grasps typically show a slowing down of the movement [i.e., increased movement time (MT) and/or a decreased peak velocity (PV) of the hand] and a reduced grip aperture [i.e., smaller maximal hand aperture (MA)] compared to normal grasps with object contact, particularly among adults that have no experience with performing pantomimed actions (Cavina–Pratesi et al. 2011; Goodale et al. 1994; Whitwell et al. 2015). The changes in kinematics are interpreted to reflect an increase in explicit or deliberate control. Deliberate control refers to the necessity to consciously attend to the way the movement is produced, which is characteristic for the control of ill-learned or novel actions (Norman and Shallice 1986). Recently, Whitwell et al. (2015a; see also Utz et al. 2015) also reported exaggerated adjustments in maximal hand aperture to object size, which may be another indication for a more deliberate control of pantomime grasps (cf. Holmes et al. 2013).

Although the described kinematic changes may point to a more conscious control mode, this in itself does not prove the involvement of the ventral system and the exploitation of allocentric sources of information. In this respect, using functional magnetic resonance imaging (fMRI), Króliczak, Cavina–Pratesi, Goodman and Culham (2007) found that pantomime grasping activated areas in the right parietal cortex; that is, the dorsal stream and not the ventral stream. On the other hand, Holmes et al. (2013) have recently demonstrated that pantomime grasping, but not normal grasping, adheres to Weber’s law. Weber’s law states that sensitivity to changes in—for instance—the size of an object is relative to its absolute size. Consequently, the smaller the object, the smaller the changes in size that an observer can still notice. Accordingly, Holmes et al. (2013) demonstrated that in pantomime grasps the within-participant standard deviation of the maximum hand aperture (i.e., a proxy for the smallest difference in object size that affects grip scaling) lessened with a decrease in object size. In contrast, no such relationship arose for normal grasps, thereby violating Weber’s law. According to the authors, this finding points to grip scaling being reliant on relative metrics during pantomime grasping, while normal grasping is guided by absolute metrics (see also Ganel et al. 2008a, b).

In the present study, we aimed to further examine the assertion that pantomime grasps engage the ventral system and exploit allocentric sources of information. To this end, we compared the effects on maximal hand aperture—the gold-standard measure in these type of studies—of presenting the to-be-grasped object within an illusory Müller–Lyer context on normal and displaced pantomimed grasps. We expected an illusory bias in the grip scaling of pantomime grasps but not the normal grasps, indicating that only pantomime grasping is influenced by the direct visual surroundings and thus relies on allocentric information.

Further to this point, Cavina–Pratesi et al. (2011) reported that the kinematic differences between displaced pantomime grasps and normal grasps were only apparent in inexperienced participants; among (professional) magicians the two grasps strongly resembled each other. Following Gonzalez, Ganel, Whitwell, Morrissey and Goodale (2008), Cavina–Pratesi et al. (2011) argued that experience in the execution of pantomiming actions leads to a shift from ventral system engagement toward stronger contributions of the dorsal system, resulting in visual control becoming less reliant on allocentric information (see also Van der Kamp et al. 2003; Willingham 1998). To directly test this proposal, we included both participants inexperienced with pantomime movements and magicians experienced with pantomime movements (Kuhn et al. 2008; MacKnik and Martinez-Conde 2011). It was hypothesised that the illusory bias in hand aperture during displaced pantomime grasping—if any—would only be present (or more pronounced) in the inexperienced participants, but not in magicians with longer experience in the execution of pantomime grasps.

## Methods

### Participants

Fourteen right-handed adults without any particular experience in pantomiming actions (age 30 ± 8 years) and eleven right-handed magicians (age 38 ± 14 years) volunteered to participate in the study. At the moment of testing, the magicians had between 2 and 46 years of experience in performing, with eight of them performing professionally. All magicians reported to use sleight-of-hand techniques that included pantomime movements (MacKnik and Martinez-Conde 2011). The local institutions’ ethical committee approved the study and all participants provided written informed consent before the start of the experiment.

### Materials and apparatus

The to-be-grasped objects were three dark-grey metal bars of 60, 80 and 100 mm in length, and 10 mm in height and width. Two additional bars of 80 mm in length had dark-grey cardboard fins attached to both ends, which were 10 mm wide and 26 mm in length. For one bar these fins pointed outward, while for the second they pointed inward, thus forming two configurations of the well-known Müller–Lyer illusion. Finally, additional bars of 40 and 120 mm in length with and without fins were used as sham objects.

**Fig. 1:**
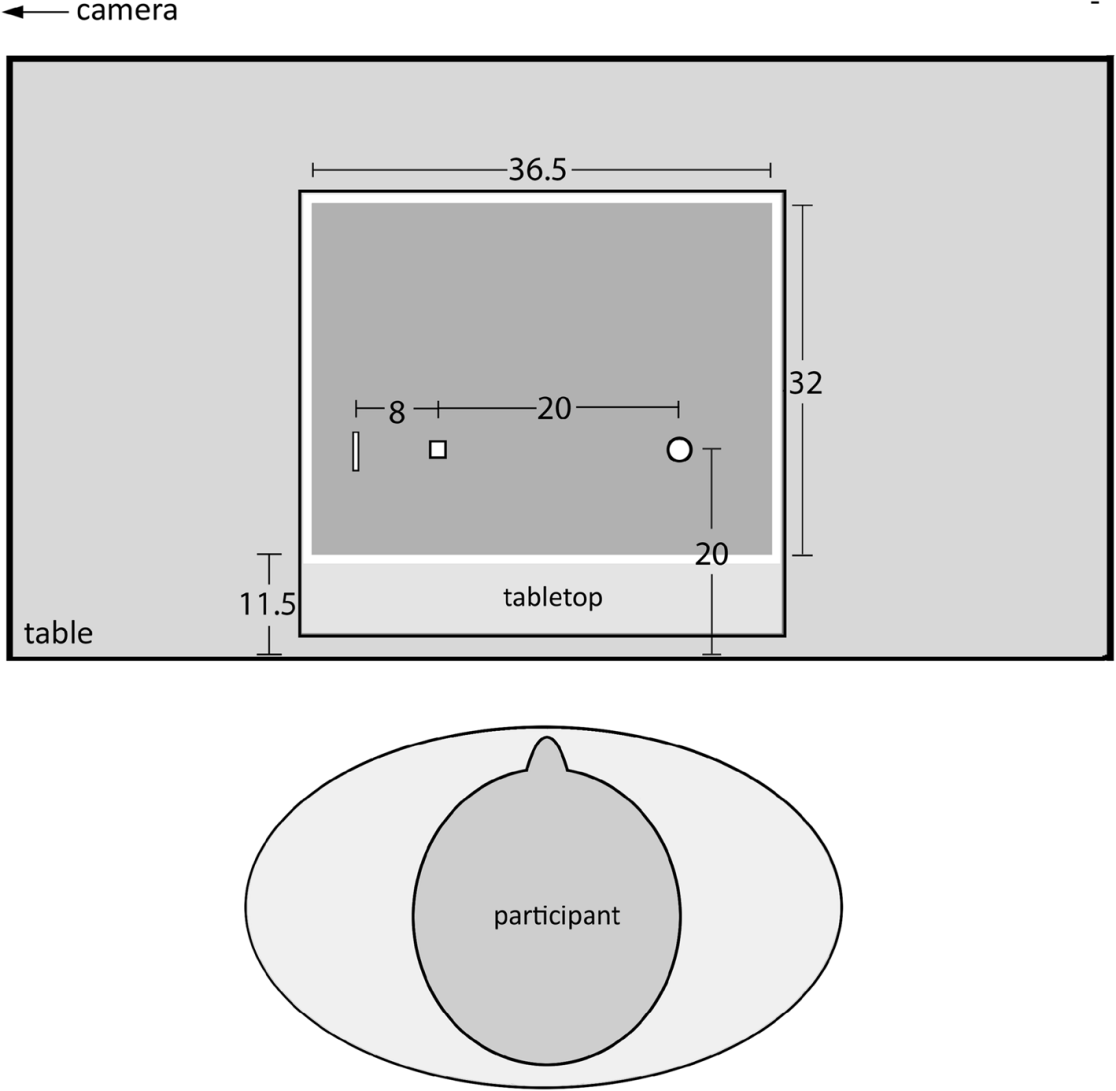
Schematic representation of the set-up (not to scale). All measures are in cm

For the normal grasping task, the objects were presented on a flat tabletop (1.15 m in height) at the participants’ approximate body midline, 30 cm from the long side of the table, where the participant was standing (see Fig. 1). The hand’s starting position was 20 cm to the right of the objects (i.e., the participants made reaches to the left in the fronto-parallel plane). For the displaced pantomime grasping task, the objects were presented 8 cm to the left of the object location during normal grasping. A small square (5 by 5 mm) drawn on the tabletop at the latter location indicated where the participants had to make the pantomimed grasp.

Finally, an Optotrak 3020 (Northern Digital Inc.) was used to record the position of a total of five infrared light emitting diodes (IREDs) attached to the tips and sides of the index finger and thumb, and the left side of the wrist of the right hand. The IREDs were sampled at a frequency of 200 Hz.

### Procedure and experimental design

Upon entering the lab, participants were informed (in writing and verbally) about the experimental procedures and then asked to provide consent to participate. Next, the three IREDs were attached to the participant’s grasping hand. The participants then performed six normal and six pantomime familiarization trials toward the five experimental objects and one of the sham objects. They were instructed to position the hand at the starting position with the tips of the index finger and thumb contacting each other, while the experimenter placed the object on the tabletop. Participants waited for the experimenter to verbally signal the start of the trial. For the normal grasps, the participant then had to reach for the object, pick it up along its longitudinal axis between index finger and thumb, lift it shortly, place it back on the tabletop, and then return the hand to the starting position. For the pantomimed grasps, participants had to reach, pick up and lift the (virtual) object as if it was placed at the location indicated by the small square drawn on the tabletop, 8 cm to the right of the object. They were told to take the length of the bar into account when grasping. No specific instructions were given regarding touching the tabletop. We did so in order not to artificially increase deliberate control, which in itself may enhance contributions of the ventral system (see “Discussion”). For both tasks, participants were asked to keep their gaze directed within the grasping area.

After the familiarization trials, the experimental trials started. The experimental trials were divided into two blocks of 30 normal grasps and two blocks of 30 pantomime grasps. The order of these four blocks was counterbalanced across participants. Within each block, the five objects (60, 80 and 100 mm without fins, and 80 mm with inward and outward fins) were presented five times in a random order. These experimental objects were interspersed with one of five sham objects. There was a 5- to 10-min break between blocks.

After finishing the experimental trials, participants filled in a short questionnaire regarding age, experience in presenting and attending magic performances, and handedness (i.e., Edinburgh Handedness Inventory).

### Data analysis and statistics

First, for each grasp, movement-onset and movement-end were determined using the multiple sources of information method developed by Schot, Brenner and Smeets (2011). This segmentation method entails the combined use of several objective kinematic functions to compute the probability that movement-start or movement-end has occurred at a particular instance (see Appendix). The instance with the highest probability is taken as the factual movement-onset and movement-end. Based on this segmentation, maximum hand aperture (MA, i.e., the largest separation (mm) between the index finger and thumb between movement-onset and movement-end), movement time (MT, i.e., the time (s) between movement-onset and movement-end), and peak velocity of the wrist (PV, i.e., the maximum absolute speed (mm/s) of the wrist between movement-onset and movement-end the moment of MA) were determined.

The main dependent variable was the corrected illusory bias in maximum hand aperture. This was calculated by dividing the difference between an individual’s average MAs for grasps toward 80 mm bars with fins-in and fins-out by the individual’s average MA for grasps towards the 80 mm bar without fins. Multiplying by 100% gives the uncorrected illusory bias in percentages. Yet, since MA in normal grasping and pantomime grasping has been found to scale differently to objects of different physical size (e.g., Whitwell et al. 2015; Utz et al. 2015), we corrected the percentage of illusory bias by dividing it by the slope of the regression line fitted through the individual’s average MAs for 60, 80 and 100 mm bars without fins (Franz et al. 2001; Stöttinger et al. 2010). The slopes were calculated using a least-squares regression method.

To compare differences in the kinematics of normal and pantomime grasps, we planned to submit MA, MT and PV to separate 2 (group: inexperienced participants, magicians) by 2 (tasks: normal grasp, pantomime grasp) by 3 (object size: 60, 80, 100 mm) ANOVAs with repeated measures of the last two factors. In the case of violation of the sphericity assumption, a Greenhouse–Geisser correction to the degrees of freedom was applied and the adjusted *p* value is reported. Also partial eta-squared (η_p_^2^) was computed to determine the proportion of variability attributable to each factor or combination of factors. Post hoc analyses were performed using *t* tests with the appropriate Bonferroni corrections. To assess differences in the corrected illusory bias percentages as a function of task and experience, we planned a 2 (group: inexperienced participants, magicians) by 2 (tasks: normal grasp, pantomime grasp) ANOVAs with repeated measures on the last factor. Finally, we ran a series of Bonferroni-corrected one-sample *t* tests to verify that any illusory bias was indeed genuine (i.e., exceeded zero).

## Results

One inexperienced participant was excluded from analysis due to procedural error. For the remaining 24 participants (11 magicians and 13 inexperienced participants), a total of 25 trials (approx., 2%) was excluded, because at the onset of the trial, hand aperture exceeded 20 mm, the hand had moved before the measurement started, or because of missing data.

### Kinematics

Tables 1, 2 and 3 report maximum hand aperture (MA), movement time (MT) and peak velocity of the wrist (PV) as function of group, task and object size. Both inexperienced participants and magicians had smaller maximal hand apertures, longer movement times and lower peak wrist velocity during pantomime grasps than during normal grasps. Indeed, analyses of variance confirmed the main effects of task for MA, *F*(1, 22) = 19.9, *p* < 0.001, η_p_^2^ = 0.48, and MT, *F*(1, 22) = 40.6, *p* < 0.001, η_p_^2^ = 0.65. Because PV was not normally distributed, a Friedman test was conducted. This failed to show a significant effect for task, χ(1) = 0.67, *p* = 0.41.

**Table 1:** Maximum hand aperture (mm) as a function of task, object size and group

	Normal			Pantomime		
	60 mm	80 mm	100 mm	60 mm	80 mm	100 mm
Inexperienced	77 (2)	94 (1)	111 (1)	67 (1)	86 (1)	103 (1)
Magicians	81 (2)	97 (2)	117 (2)	73 (2)	93 (2)	113 (2)

Numbers between brackets indicate SE

**Table 2:** Movement time (s) as a function of task, object size and group

	Normal			Pantomime		
	60 mm	80 mm	100 mm	60 mm	80 mm	100 mm
Inexperienced	0.79 (0.04)	0.81 (0.05)	0.89 (0.05)	0.94 (0.06)	0.96 (0.06)	1.00 (0.07)
Magicians	0.95 (0.04)	0.98 (0.04)	1.05 (0.05)	1.20 (0.04)	1.23 (0.04)	1.26 (0.03)

Numbers between brackets indicate SE

**Table 3:** Peak velocity (mm/s) as a function of task, object size and group

	Normal			Pantomime		
	60 mm	80 mm	100 mm	60 mm	80 mm	100 mm
Inexperienced	488 (24)	493 (23)	501 (23)	473 (19)	473 (22)	484 (24)
Magicians	423 (18)	0.425 (14)	439 (17)	403 (15)	412 (17)	426 (14)

Numbers between brackets indicate SE

In addition, analyses of variance (for MA and MT) and a Mann–Whitney *U* test (for PV) confirmed significant effects of group for MA, *F*(1, 22) = 13.9, *p* < 0.01, η_p_^2^= 0.39, MT, *F*(1, 22) = 11.6, *p* < 0.001, η_p_^2^= 0.35, and PV, *Z* = −2.4, *U* = 30.0, *p* < 0.05. As can be seen from Tables 1, 2 and 3, on average, magicians had larger MA, longer MT and lower PV. To explore whether these effects are merely reflecting hand size,^2^ an additional independent *t* test was performed. This, however, did not reveal a significant difference, *t*(22) = −1.24, *p* = 0.24, *r* ^2^ = 0.03. Finally, no interactions between task and group were present.

Tables 1, 2 and 3 also illustrate that both groups of participants adjusted hand aperture, movement duration, and peak wrist velocity to the size of the bars. Accordingly, analyses of variance and a Friedman test showed main effects of object size for MA, *F*(2, 44) = 2472.0, *p* < 0.001, η_p_^2^= 0.99, MT, *F*(2, 44) = 47.4, *p* < 0.001, η_p_^2^ =0.68, and for PV, χ(1) = 9.3, *p* < 0.05. Moreover, object size significantly interacted with task for MA, *F*(2, 44) = 10.5, *p* < 0.001, η_p_^2^= 0.32, and MT, *F*(2, 44) = 3.6, *p* < 0.05, η_p_^2^ = 0.14. Post hoc analysis indicated MA was smaller during pantomime than during normal grasps for the 60 mm bars, suggesting that adjustments of the hand aperture to object size were somewhat larger for pantomime grasping. In fact, a direct comparison of grip scaling in the two tasks shows that the linear slope relating MA to object size was significantly steeper for pantomime grasping (*M* = 0.95, SD = 0.12) than for normal grasping (*M* = 0.86, SD = 0.09), *t*(24) = −3.34, *p* < 0.01. Conversely, adjustments in MT to object size appeared larger for normal grasps: that is, post hoc analysis indicated significantly longer MT for the 100 mm than 60 mm bars for both grasps, but the difference between the 80 mm and 60 mm bar was only significant for the normal grasps. With respect to PV, Wilcoxon signed-rank tests indicated an increased PV for the 100 mm bar as compared to the 80 and 60 mm bars. This was observed for both pantomime and normal grasps. Finally, for MA a significant object size by group interaction was revealed, *F*(2, 44) = 4.5, *p* < 0.05, η_p_^2^= 0.17. Yet, post hoc analyses did not result in localizing the source of the interaction. We also tested for differences in grip scaling between the two groups by comparing the linear slopes relating MA to object size. This revealed slightly steeper slopes for magicians (*M* = 0.94, SD = 0.10) than for the inexperienced control participants (*M* = 0.88, SD = 0.07). However, this difference was not significant, *t*(22) = −1.87, *p* = 0.08.

### Illusory bias

**Fig. 2:**
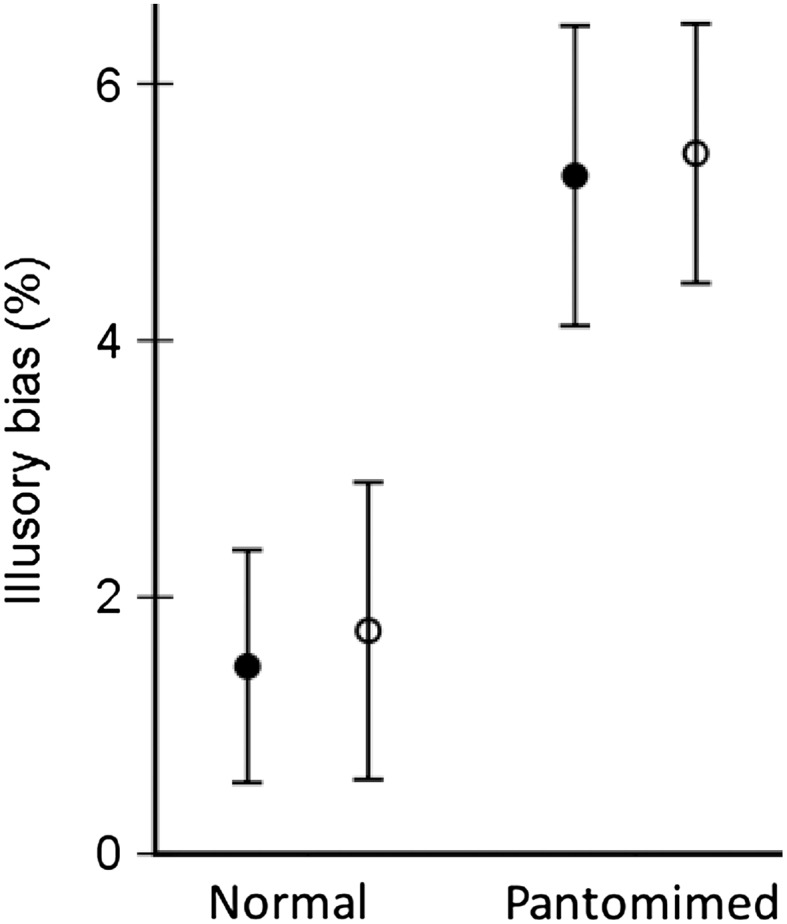
Percentage illusory bias as a function of task and group. *Filled circles* represent inexperienced participants, while *open circles* represent experienced magicians. *Error bars* represent SE

**Fig. 3:**
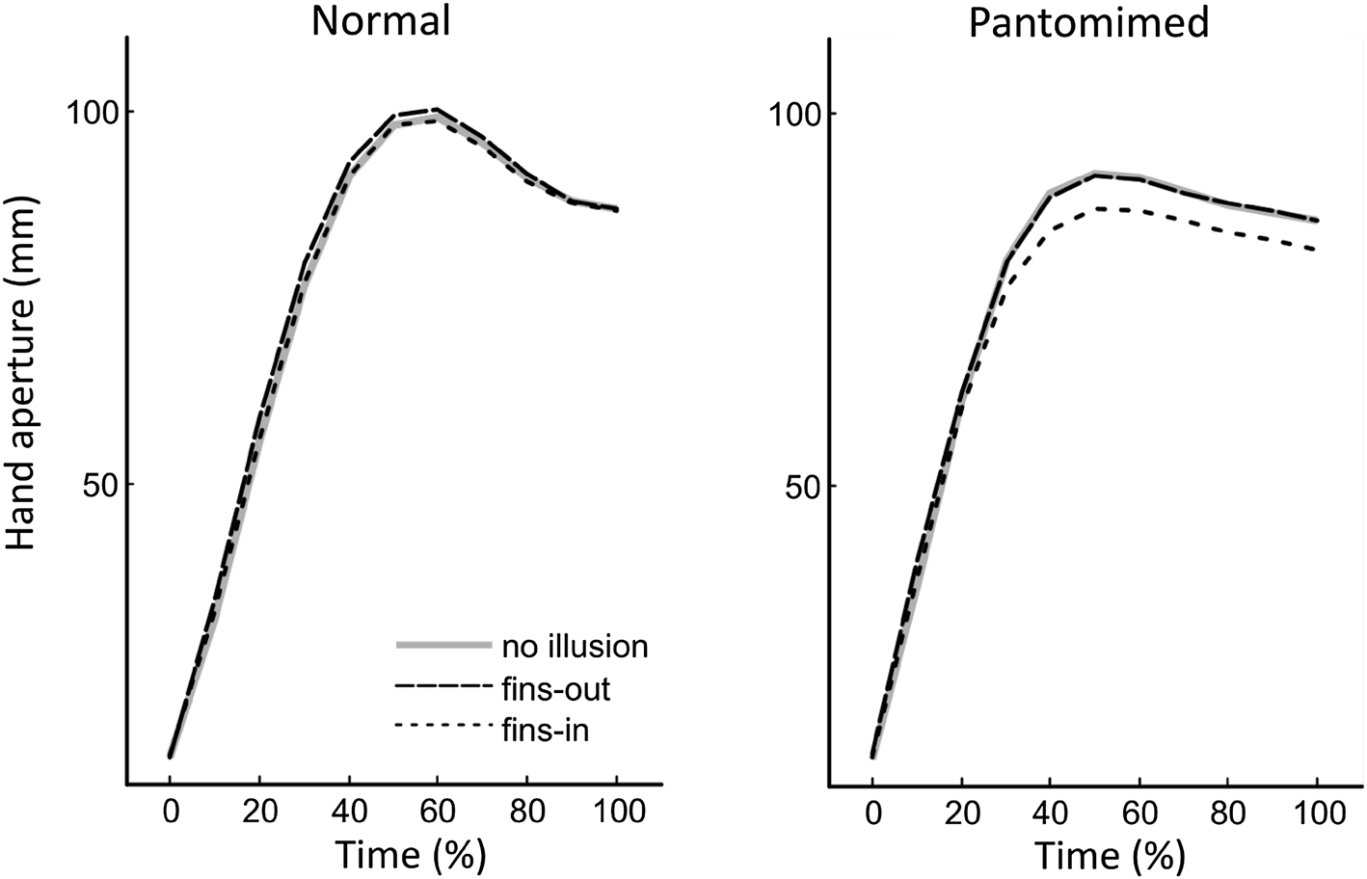
Time-normalised profiles of hand aperture as a function of configuration non-illusory object (*plain lines*), fins-in (*dotted lines*) and fins-out (*dashed lines*) for the 80 mm *bars. Left panel* normal grasping; *right panel* displaced pantomime grasping

The time-normalised hand aperture profiles in Fig. 3 illustrate how the illusory bias unfolds over time. Clearly, the illusory bias was larger for the pantomime grasp than for the normal grasp, although the bias in pantomiming mainly derives from the fins-in configuration. The most pertinent variable is the percentage of corrected illusory bias in maximal hand aperture. This is displayed in Fig. 2 as a function of task and group (see also Table 4). The analysis of variance confirmed a significant effect of task, *F*(1,22) = 17.0, *p* < 0.001, η^2^ = 0.44, but did not return significant effects for group and group by task. Subsequently, a series of Bonferroni-corrected one-sample *t* tests confirmed that for both groups the illusory biases in the pantomime grasps were larger than zero, *t*’s > 4.5, *p*’s < 0.001, while the bias did not exceed zero for the normal grasps, *t*’s < 1.61, *p*’s > 0.13.

**Table 4:** Uncorrected and corrected illusory biases as a function of task and group

	Uncorrected		Corrected	
	Normal	Pantomimed	Normal	Pantomimed
Inexperienced	1.16 (2.57)	4.79 (3.86)	1.46 (3.27)	5.29 (4.22)
Magicians	1.67 (3.59)	5.52 (3.62)	1.74 (3.85)	5.46 (3.35)

Numbers between brackets indicate SD

### Effects of pantomime experience

It may be hypothesised that the magnitude of the differences between maximal hand apertures for normal and pantomime grasps reflects pantomime skill: the smaller the difference, the better the participant is capable of mimicking a normal grasp. The magicians had an average experience of performing of 21.6 (±14.6) years. Although the correlation between experience and the difference in MA between the two tasks was moderately high, it was not significant, Pearson *r* = 0.56, *p* = 0.08. In fact, a high Cook’s distance (*D* = 1.18) indicated that one data point likely has a disproportionate influence on the correlation coefficient. Hence, this finding must not be over-interpreted. Next, we explored whether the frequency of magicians’ performances would be an indicator of the magicians’ pantomime skills. On average the magicians performed 55.1 (±39.7) times during a year. Because the frequency of performances was not-normally distributed, Kendall’s tau was used. This showed that the more frequently a magician performed, the smaller the difference in MA between the two tasks, Kendall τ = −0.50, *p* = 0.034. However, neither years of experience, Pearson *r* = −0.14, *p* = 0.69, nor frequency of performances, Kendall τ = −0.31, *p* = 0.18, was correlated to illusory bias.

## Discussion

The present study examined whether normal and pantomimed grasps exploit different sources of information. That is, according to a recent amendment to Milner and Goodale’s two-visual-system model, normal actions towards tangible objects engage the dorsal system and primarily rely on egocentric information, but only if the action results in tactile contact with the target object (Whitwell et al. 2014). Without contact, however, the ventral system would get involved and allocentric information will be used in the control of the action. We specifically addressed the latter conjecture. Displaced pantomime grasps, which entail performing grasps next to a visible object without making contact, were hypothesised to involve enhanced reliance on allocentric information as compared to normal grasps. We tested this by having magicians experienced with pantomime movements and participants without any particular pantomime experience perform displaced pantomime grasps and normal grasps toward objects that were embedded in an optic illusion. We presumed that the use of allocentric sources of information would induce an illusory bias in the grasping movements (Ganel et al. 2008a, b; Stöttinger et al. 2010). We further examined if experience in pantomime would reduce the bias or whether the bias would persist with experience (Cavina–Pratesi et al. 2011).

The participants showed kinematically distinct movement patterns when performing normal and pantomime grasps. For pantomime grasps, hand aperture reduced, movement time increased, and peak wrist velocity tended to be lower than in normal grasps, although the latter was not significant. These differences were observed both among the experienced magicians and the inexperienced participants. In fact, the nature of these differences resembles the kinematic differences reported in previous studies (e.g., Cavina–Pratesi et al. 2011; Goodale et al. 1994; Holmes et al. 2013; Whitwell et al. 2015a). The observed reduced movement time and lower wrist velocity for the pantomime grasp are typically interpreted as reflecting conscious or deliberate control, while the reduced hand aperture is thought to reflect the loss of the constraint to exceed or overshoot object size (see Westwood and Goodale 2003; Wing et al. 1986). Yet, the present study also suggests that adjustments in hand aperture to object size were somewhat exaggerated in pantomime grasps as compared to real grasps, which may also point to increased deliberate control (Utz et al. 2015; Whitwell et al. 2015a).

A more deliberate control has often been associated with increased engagement of the ventral system (e.g., Rossetti 1998; Gonzalez et al. 2008; Willingham 1998). Hence, the observed differences in kinematics between pantomime and normal grasps have been interpreted as revealing the predominance of the ventral system’s involvement in pantomime grasping (Goodale et al. 1994; Cavina–Pratesi et al. 2011). Nevertheless, at best, the kinematic differences provide circumstantial evidence for the ventral system’s engagement. Westwood et al. (2000), however, reported that the bias induced by an illusory context is enhanced for pantomime grasps as compared to normal grasps. Westwood et al., however, used a delayed pantomime task, during which participants grasp toward remembered objects. We replicated this finding for a displaced pantomime task during which the object and its visual surroundings are in full view while performing the pantomime. The presence of an illusory bias in displaced pantomime grasping but not in normal grasping in the present study does lend further credence to the engagement of the ventral system; without tactile contact with the goal object, movement control comes to rely more strongly on allocentric information. This supports the recent amendment to the two-visual-system model that tactile contact with the goal object is a prerequisite for the dorsal system to be implicated in the control of action or else the ventral system gets involved (Whitwell et al. 2014).

However, there may be reasons for tempering this claim. Unexpectedly, the illusory bias in the pantomime grasp was observed to be limited to the fins-in configuration of the Müller–Lyer illusion; the fins-out configuration did not noticeably influence hand aperture (Fig. 3). Because the majority of studies only report the bias, it is unclear how unique this asymmetry is. One reason may be that moderately large goal objects (i.e., 80 mm) compromised the magnitude of the illusion, because the hand cannot be further enlarged due to physical constraints. This account, however, appears to be invalidated by the larger hand apertures for the normal grasps. Conversely, the same constraint may have thwarted any illusory bias for the fins-out configuration in normal grasping. Yet, if true, then a bias in normal grasping should have emerged for the fins-in configuration, but it did not. A second perhaps more pertinent issue is that participants were not prevented to make contact with the table surface to complete the action. Whitwell et al. (2014) argued that for patient DF, tactile contact with the direct surroundings of the object could have acted as a proxy for tactile contact with the object itself, thereby circumventing engagement of the ventral system. Indeed, they showed that haptic feedback at the end of the action per se, rather than haptic information on object size supported grip scaling in patient DF (see also Whitwell et al. 2015b). The current participants may have contacted the tabletop in approximately half of the trials (i.e., in these trials the minimum distance with the table surface of the IREDs attached to the index finger and/or thumb at the end of the grasp was smaller than the minimum distance at the starting location before reach onset). Nonetheless, the illusory bias did emerge in pantomime grasping. We suggest therefore that in neurologically healthy participants, it is the lack of tactile contact with the goal object rather than terminal haptic feedback that appears to induce the ventral system engagement. This may or may not have been augmented by the locations of the viewed object and the action being incongruent.

The illusory bias in pantomime grasping was of equal magnitude in the inexperienced participants and the experienced magicians. This contrasts with the suggestion by Cavina–Pratesi et al. (2011) that with experience the control of pantomime grasping shifts from the ventral to the dorsal system. Cavina–Pratesi et al. (2011) based their contention of a shift on the observation that the kinematic differences between pantomime and normal grasps in novice participants had vanished among the magicians. In the current study, we indeed found that maximal hand aperture and movement time of the pantomime grasps were larger for the magicians as compared to the inexperienced participants, but these kinematic differences also emerged for the normal grasps. This might reflect exaggeration or a high degree of deliberate control among magicians both for the pantomime and the normal grasps, but additional tests, such as using a dual-task paradigm, will be needed to further assess this speculation. Importantly, however, it raises the issue whether the magicians were genuinely experts in pantomime. If sleight of hand does not necessarily translate into pantomime skill, then we must be cautious in rejecting Cavina–Pratesi et al.’s contention of a skill-related shift from ventral to dorsal control in pantomiming. This being said, we do find that within the groups of magicians the number of performances (but not the years of experience as a magician) relates to the magnitude of the kinematic differences between pantomime and normal grasps. This implies that not all the magicians were unskilled in pantomime actions. Moreover, whatever the source of this relationship, it is unlikely to point to a reduced engagement of the ventral system with increases in pantomime skill, since both the number of magical performances and years of experience did not correlate with the illusory bias in the pantomime grasps.

In conclusion, the present study supports the notion that the control of pantomimed grasps performed next to the visible goal object relies on allocentric or world-centred information and thus involves the ventral system. Moreover, the evidence indicates that this reliance persists with experience. Importantly, these observations support a recent addition to the two-visual-system model that the normal engagement of the dorsal system requires tactile contact with a goal object. If no tactile contact is made, and the goal object is in full view, then control shifts toward the ventral system.

## Appendix

Following Schot et al. (2011), the “multiple sources of information” method was used to determine movement-onset and movement-end. This method defines a series of objective functions that together represent the likelihood that movement-onset and movement-end occur at each instance of time. That is, for each of the functions, at each instant in time, a likelihood value is assigned that corresponds to movement-onset and movement-end. The values range from 0 to 1, with larger values representing a higher chance that movement-onset and movement-end occurred. The multiplication of the values of all the objective functions results in a time-series. The instance in time with the highest value (i.e., the maximum likelihood) was taken as movement-onset and movement-end.

### Movement onset

Six objective functions for the start of the movement were defined.

1. The time (t) elapsed is less than 2 s.
  1 $$t\text{ }<\text{ }2.0\text{ }=\text{ }1\quad \text{and}\quad t\text{ }>\text{ }2.0\text{ }=\text{ }0.$$
2. The index finger (*I*) and the thumb (*T*) moved over a distance of not more than 15 mm from the starting position, both in the direction of the object (*D*_y_) as well as upwards (*D*_z_).
  2 $${\text{IDy }} \& {\text{ IDz }} \& {\text{ TDy }} \& {\text{ TDz }} < 15 = 1 {\text{ and IDy }}\& {\text{ IDz }}\& {\text{ TDy }}\&{\text{ TDz}}> 15 = 0.$$
3. MA is equal to or smaller than 20 mm.
  3 $$\text{MA}\le 20\text{ }=1\text{ and MA}>\text{ }20\text{ }=0.$$
4. Hand velocity (*V*) should be low, but not zero. We defined hand velocity as the average between the velocities of the index finger, thumb and wrist both in the direction of the object (avV_y_) as well as upward (avV_z_). Next, these were converted into continuous ranges between 0 and 1 by dividing it with their maximums (i.e., avVrel_y,_ avVrel_z_). A peak-like function was then obtained by subtracting these relative hand velocities from their squared roots. Because at the start of the movement the relative velocity in the direction of the object and upward must exceed 0, the two relative hand velocities were multiplied.
  4 $$\left(\sqrt {avVrel_{y} }- {\text{ avVrely }}\right) \times \left({\text{ }}\sqrt {avVrel_{z} } -{\text{ avVrelz}}\right){\text{.}}$$
5. When the index finger (*I*), thumb (*T*) and wrist (*W*) start to move and both the distance between starting point and the object in the direction of the object (*D*_y_) decrease and the distance in the upward direction (*D*_z_) increases, then this indicates that movement has started. First, the distances were converted into continuous ranges between 0 and 1 by dividing them through their maximums (IDrel_y_, IDrel_z_), and then subtracted from 1 (i.e., the closer the value is to 1, the nearer to movement onset) and multiplied.
  5 $$\left(1\text{ }-\text{ }\left( \text{IDre}{{\text{l}}_{y}} \right)\right)\text{ }\times \text{ }\left(1-\text{ }\left( \text{IDre}{{\text{l}}_{z}} \right)\right)$$
  6 $$\left(1-\left( \text{TDre}{{\text{l}}_{y}} \right)\right)\text{ }\times \text{ }\left(1-\text{ }\left(\text{TDre}{{\text{l}}_{z}}\right)\right)$$
  7 $$\left(1\text{ }-\text{ }\left( \text{WDre}{{\text{l}}_{y}} \right)\right)\text{ }\times \text{ }\left(1-\text{ }\left( \text{WDre}{{\text{l}}_{z}} \right)\right)$$
6. An increase in the rate of change of hand aperture (AV, i.e., opening velocity) indicates that the hand starts to open and the movement has started. To create a continuous range between 0 and 1, we divided the rate of change of hand aperture by its maximum AVmax.
  8 $$\text{(AV}/\text{ }\left( \text{AVmax} \right))$$

### Movement end

Movement end was defined as the instance in time the object is lifted. Six objective functions were defined.

1. The movement ends in the time interval between the instance that MA is reached (tMA) and the instance that the hand is at its highest point (i.e., after tMa = 250 ms) but not later than 4.75 s. The maximum of the average of the index finger (I) and thumb (T) in the upward direction (avPmax_z_) was defined as the hand’s highest point.
  9 $$\text{tMA}<\text{ }t\text{ }<\text{ tavPma}{{\text{x}}_{z}}=1\text{ or else }0$$
2. The movement ends when the index finger (*I*) and thumb (*T*) are positioned at least 190 mm from the starting position in the direction towards the object (*P*_y_) and 20 mm or less above the table top (*P*_z_).
  10 $${\text{IPy \& TPy> 190 and IPz \& TPz < 20 = 1 or else 0}}$$
3. The thumb and index finger do not contact each other while grasping the object, and should have some minimum value.
  11 $$\text{MA}\ge 45=1\text{ and MA }<\text{ }45=0$$
4. Wrist velocity (*W*) in the direction towards the object (*V*_y_) should be at minimum. To this end, the wrist velocity relative to its maximum PV_y_ was computed (i.e., WVrel_y_) and subtracted from 1.
  12 $$1{\text{ }}{-}{\text{ WVRel}}_{y}$$
5. The hand should just be starting to move upwards (i.e., lifting the object). Hence, we took the average of the velocities of the index finger (*I*), thumb (*T*) and wrist (*W*) in the upward direction (avV_z_), and converted this into a continuous range between 0 and 1 by dividing it through maximum hand speed (PV_z_). A peak-like function was then obtained by subtracting this relative hand velocity (i.e., avVrel_z_) from its squared root, which cannot become zero.
  13 $$~\sqrt {{\text{avVrel}}_{z} } {-}{\text{ avVrel}}_{z}$$
6. If—after tMA—the rate of change of hand aperture approaches zero (AV, i.e., closing velocity), then the object is contacted and about to be lifted, signalling movement end. To create a continuous range between 0 and 1, we computed the absolute of the rate of change of hand aperture divided by its maximum (AVmax) and subtracted it from 1.
  14 $$1\text{ }-\text{ }\left| \text{ AVrel } \right|$$

## Footnotes

1 The present study exclusively relies on behavioural observations; no brain-imaging techniques were used to measure activity of the ventral and dorsal streams. For this reason, ‘ventral and dorsal *systems’*, as we use here, must be primarily understood as functional systems for the detection and use of visual information for perceiving or acting, respectively. When we refer to the underlying neural circuitry, we use ‘ventral and dorsal *streams’*.

2 To explore whether magicians had larger hands than the inexperienced control participants, we estimated participants’ hand sizes improviso after the completion of the experiment using the grasp kinematics. We reasoned that the largest distance between the top of the index finger and wrist (i.e., when the index finger reaches maximum extension) would be correlated to hand size. Hence, we calculated the average distance between the IREDs attached to the index finger and the wrist at the moment of maximal hand aperture (MA) for normal grasps directed to the largest 100 mm bar for each individual participant. Rather than including all trials, the average distance was based on trials with a MA that exceeded the individual participant’s median MA for normal grasps directed to the 100 mm bar.
